# Biodegradation of endocrine disruptor Bisphenol A by *Pseudomonas putida* strain YC-AE1 isolated from polluted soil, Guangdong, China

**DOI:** 10.1186/s12866-020-1699-9

**Published:** 2020-01-13

**Authors:** Adel Eltoukhy, Yang Jia, Ruth Nahurira, M. A. Abo-Kadoum, Ibatsam Khokhar, Junhuan Wang, Yanchun Yan

**Affiliations:** 1 Graduate School of Chinese Academy of Agricultural Sciences, Beijing, 100081 China; 2 Botany and Microbiology Department, Faculty of Science, AL-Azhar University, Assiut, 71524 Egypt; 3 Institute of Modern Biopharmaceuticals, School of Life Sciences, Southwest University, Chongqing, 400715 China

**Keywords:** Bisphenol A, Biodegradation, *Pseudomonas putida*, Endocrine disruptor, Degradation pathway, Response surface methodology

## Abstract

**Background:**

Bisphenol A is an important organic chemical as an intermediate, final and inert ingredient in manufacturing of many important products like polycarbonate plastics, epoxy resins, flame retardants, food–drink packaging coating, and other. BPA is an endocrine disruptor compound that mimics the function of estrogen causing damage to reproductive organs. Bacterial degradation has been consider as a cost effective and eco-friendly method for BPA degradation compared with physical and chemical methods. This study aimed to isolate and identify bacterial strain capable to degrade and tolerate high concentrations of this pollutant, studying the factors affecting the degradation process and study the degradation mechanism of this strain.

**Results:**

YC-AE1 is a Gram negative bacterial strain isolated from soil and identified as *Pseudomonas putida* by 16S rRNA gene sequence and BIOLOG identification system. This strain found to have a high capacity to degrade the endocrine disruptor Bisphenol A (BPA). Response surface methodology using central composite design was used to statistically optimize the environmental factors during BPA degradation and the results obtained by significant model were 7.2, 30 °C and 2.5% for optimum initial pH, temperature and inoculum size, respectively. Prolonged incubation period with low NaCl concentration improve the biodegradation of BPA. Analysis of variance (ANOVA) showed high coefficient of determination, *R*^*2*^ and *Adj-R*^*2*^ which were 0.9979 and 0.9935, respectively. Substrate analysis found that, strain YC-AE1 could degrade a wide variety of bisphenol A-related pollutants such as bisphenol B, bisphenol F, bisphenol S, Dibutyl phthalate, Diethylhexyl phthalate and Diethyl phthalate in varying proportion. *Pseudomonas putida* YC-AE1 showed high ability to degrade a wide range of BPA concentrations (0.5–1000 mg l^− 1^) with completely degradation for 500 mg l^− 1^ within 72 h. Metabolic intermediates detected in this study by HPLC-MS were identified as 4,4-dihydroxy-alpha-methylstilbene, *p*-hydroxybenzaldeyde, *p*-hydroxyacetophenone, 4-hydroxyphenylacetate, 4-hydroxyphenacyl alcohol, 2,2-bis(4-hydroxyphenyl)-1-propanol, 1,2-bis(4-hydroxyphenyl)-2-propanol and 2,2-bis(4-hydroxyphenyl) propanoate.

**Conclusions:**

This study reports *Pseudomonas putida* YC-AE1 as BPA biodegrader with high performance in degradation and tolerance to high BPA concentration. It exhibited strong degradation capacity and prominent adaptability towards a wide range of environmental conditions. Moreover, it degrades BPA in a short time via two different degradation pathways.

## Background

Xenobiotic endocrine disruptor compounds are environmental pollutants that have health and environmental harms, raising important concerns [1, 2]. Bisphenol A (BPA), 2,2-bis(4-hydroxyphenyl) propane is a well-known endocrine disruptor that is showing a highly estrogenic activity and acute toxicity [3]. It can bind to receptors for other hormonally mediated processes and cause endocrine disruptions. EPA and the U.S. Food and Drug Administration established a safe reference dose (RfD) for humans at 50 μg/kg/day [4]. Since hormones levels in the human body are present in biologically active concentrations, exposure to these exogenous chemicals even in low doses can disrupt the proper functioning of the body’s endocrine system. In addition, dose scaling is valid for agents that follow linear dose-response relationships, many endocrine-disruptors, like their hormonal counterparts, demonstrate a non-monotonic dose-response curve and in this case, lower doses are as relevant as higher doses [5–7]. Chemically, BPA is an organic compound consisting of two phenolic rings connected by a carbon bridge with two methyl group attached to this bridge [8].

The production of BPA has increased in the last few decades because of its widespread application in many industries [9] to reach the high global demand which was approximately 6.5 million tons in 2012 [10, 11]. BPA is used as a major component (as plasticizer) for various consumer products including detergents, phenol resins, epoxy resins, polycarbonates, polyesters, polyacrylates, plastic packing materials and lacquer coatings on food cans [11]. There are large number of studies that reported many damaging effects of BPA for both human and animal organs [12]. Detectable concentrations of BPA were reported in amniotic fluid, fetal circulation and placental tissue of pregnant women [13]. Many studies reported that, exposure of aquatic animals to BPA cause reproductive toxicity like changing in sex hormone level which affected the development of ovary in rare minnow *Gobiocypris rarus* [14], decreased sperm count, female biased sex ratio, and increased malformation and mortality in zebra fish [15]. BPA was detected in a wide range of environments including landfill leachates [16], industrial wastewater [17], source water and drinking water [18], rivers, seas, and soils [19–22]. Therefore, special attention has been given to BPA toxicity in these environments [23]. The most common methods to remove pollutants from the environment included photodegradation, photoelectrocatalytic oxidation, oxidation [17] and biodegradation [24]. Among all these methods, bacterial biodegradation of BPA was the most important one [25] because of low costs and less disruption of the contaminated environment when compared to other cleanup methods. Many researchers reported several BPA-degrading bacteria and their degrading performance *Achromobacter xylosoxidans* strain B-16 [25], *Pseudomonas* sp. and *Pseudomonas putida* strain [26], *Bacillus megaterium* Strain ISO-2 [27], *Sphingomonas bisphenolicum* strain AO1 [28] and *Pseudomonas* sp. and *Sphingomonas* sp. [10].

Microorganisms and their products are very important in industrial processes such as in synthesis of a number of products and fermentation processes. Their use is influenced by environmental factors such as a temperature, pH, nutrients and as must be prior optimized before application. The optimization by one factor at time (OFAT) involves changing in one factor while other factors or variables at certain levels are fixed [29]. This method of optimization is time consuming especially with large number of variables in addition to, ignoring the interactions between different variables. These disadvantages of single-factor experiments can be overcome by using statistical optimization which allows all interactions of all variables and levels [30, 31].

In this study, a highly BPA tolerant strain with high BPA biodegradation capacity was isolated from soil sample beside the electronic wastes collection area in Guangdong province, China and identified as *Pseudomonas putida* YC-AE1, the environmental conditions affecting BPA degradation were studied using statistical methods and discussed. The ability of strain YC-AE1 to degrade other pollutants related to BPA was also studied. The metabolic intermediate compounds detected during the biodegradation process were identified and biodegradation pathways were proposed.

## Results

### Isolation and identification of BPA degrading strain

**Fig. 1:**
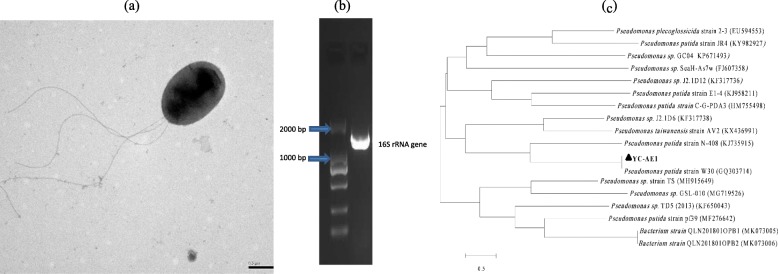
Identification of YC-AE1, (**a**) TEM investigation showing cell shape and lophotrichous flaggelation (**b**) amplified 16S rRNA gene (about 1500 bp) comparing with 2000 bp DNA ladder, (**c**) phylogenetic tree of *Pseudomonas putida* YC-AE1 with closely related strains

YC-AE1 strain isolated from the soil sample of Guangdong province showed high degradation and tolerance to BPA. It is a rod-shaped, flagellated, Gram-negative bacterium. The amplified 16S rRNA gene sequence (1439 bp) was deposited in Gen Bank (accession number, MK318658) and related strains were obtained by BLAST. The similarity between YC-AE1 and others was detected by constructing a phylogenetic tree (Fig.1) and the most closely related one was *Pseudomonas putida* strain W30 with 99% similarity. The BIOLOG tests was performed and showed consistent results with the 16S rRNA gene (PROB = 97.2%, SIM = 0.849, and DIST = 1.818 with *Pseudomonas putida*). According to morphology, BIOLOG tests and 16S rRNA gene sequencing, our strain YC-AE1 identified as *Pseudomonas putida*.

### Statistical optimization of BPA degradation using RSM

The combined five factors with their levels and obtained experimental results are presented in Table 1, the highest BPA degradation (95%) was observed in the result obtained by the design (run No. 21). The design was analyzed by regression analysis resulted in a significant model with the following regression equation:

$$ {\displaystyle \begin{array}{l} BPA\; degradation=77.93957+14.45A-12.65B+22C+11.425D-4.45E+\\ {}1.38625 AB-1.48625 AE-2.71125 BD+17.65125 BE-11.9997{A}^2-3.59973{B}^2-\\ {}8.27473{C}^2-9.68723{D}^2-17.5497{E}^2-7.42375{A}^2B-21.6013{A}^2C-8.23875{A}^2D-\\ {}18.7013{A}^2E-13.8863A{B}^2\end{array}} $$

**Table 1:** Experimental design (conditions and responses) for BPA degradation by *Pseudomonas putida* YC-AE1

Run No.	A	B	C	D	E	BPA degradation (%)^a^
	Initial pH (values)	NaCl Conc. (%)	Incubation period (hours)	Inoculum size (%)	Incubation temp. (°C)	
7	5.00 (−2)	0.75 (0)	20 (0)	2.5 (0)	31 (0)	2.2
5	6.13 (−1)	1.13 (+ 1)	15 (− 1)	3.25 (+ 1)	37 (+ 1)	2.7
8	6.13 (−1)	1.13 (+ 1)	15 (−1)	1.75 (− 1)	25 (− 1)	7.8
10	6.13 (−1)	0.38 (− 1)	25 (+ 1)	1.75 (− 1)	25 (− 1)	81.7
11	6.13(− 1)	1.13 (+ 1)	25 (+ 1)	1.75 (− 1)	37 (+ 1)	0
12	6.13 (−1)	0.38 (− 1)	15 (− 1)	1.75 (− 1)	37 (+ 1)	0
14	6.13 (− 1)	1.13 (+ 1)	25 (+ 1)	3.25 (+ 1)	25 (− 1)	6.4
28	6.13 (−1)	0.38 (− 1)	25 (+ 1)	3.25 (+ 1)	37 (+ 1)	13.38
29	6.13 (−1)	0.38 (− 1)	15 (− 1)	3.25 (+ 1)	25 (− 1)	93.5
1	7.25 (0)	0.75 (0)	10 (−2)	2.5 (0)	31 (0)	2
2	7.25 (0)	1.5 (+ 2)	20 (0)	2.5 (0)	31 (0)	39.4
3	7.25 (0)	0.75 (0)	20 (0)	2.5 (0)	31 (0)	77
4	7.25 (0)	0.75 (0)	20 (0)	2.5 (0)	19 (−2)	17.8
15	7.25 (0)	0.75 (0)	20 (0)	2.5 (0)	31 (0)	77.2
16	7.25 (0)	0.75 (0)	30 (+ 2)	2.5 (0)	31 (0)	90
19	7.25 (0)	0.75 (0)	20 (0)	4 (+ 2)	31 (0)	63.2
20	7.25 (0)	0 (−2)	20 (0)	2.5 (0)	31 (0)	90
22	7.25 (0)	0.75 (0)	20 (0)	2.5 (0)	31 (0)	77.3
23	7.25 (0)	0.75 (0)	20 (0)	2.5 (0)	43 (+ 2)	0
24	7.25 (0)	0.75 (0)	20 (0)	1 (−2)	31 (0)	17.5
6	8.38 (+ 1)	0.38 (−1)	25 (+ 1)	1.75 (−1)	37 (+ 1)	1.7
9	8.38 (+ 1)	1.13 (+ 1)	15 (−1)	3.25 (+ 1)	25 (− 1)	17.5
13	8.38(+ 1)	1.13 (+ 1)	25 (+ 1)	1.75 (−1)	25 (− 1)	15
18	8.38(+ 1)	0.38 (−1)	15 (−1)	3.25 (+ 1)	37 (+ 1)	7
21	8.38 (+ 1)	0.38 (−1)	25 (+ 1)	3.25 (+ 1)	25 (− 1)	95
25	8.38 (+ 1)	1.13 (+ 1)	15 (−1)	1.75 (− 1)	37 (+ 1)	0
26	8.38 (+ 1)	1.13 (+ 1)	25 (+ 1)	3.25 (+ 1)	37 (+ 1)	0
27	8.38 (+ 1)	0.38 (−1)	15 (− 1)	1.75 (− 1)	25 (− 1)	78.3
17	9.5 (+ 2)	0.75 (0)	20 (0)	2.5 (0)	31 (0)	60

^a^All response (BPA degradation percentage) values are shown as means for three independent experiments

where, A, B, C, D and E are the actual values of initial pH, NaCl concentration, incubation period, inoculum size and Incubation temperature, respectively. Regression analysis for the biodegradation shown in Table 2, indicate that the model used in our study was highly significant with very low *P*-value (0.0001) and high F-value (229.53). As shown in Table 2, all linear and quadratic variables were highly significant with *p*-value close to (0.00) indicating the model success. Two of variable interactions; pH and NaCl concentration (AB) and pH and incubation temperature (AE) were slightly non-significant with *p*-values 0.0944 and 0.0760 respectively, indicating the interactions were not favorable to the response while, the other two variables, NaCl concentration and inoculum size (BD) and NaCl concentration and incubation temperature (BE) were highly significant. The model fitness was expressed by the coefficient of determination, R^2^ and Adj-R^2^ which were 0.9979 and 0.9935, respectively.

**Table 2:** Regression analysis for the biodegradation of BPA by *Pseudomonas putida* YC-AE1

Source	Coefficient	Sum of Squares	Degrees of freedom	Mean Square	F Value	*P* value Prob > F
Model		38,370.26	19	2019.48	229.53	< 0.0001
pH, A	14.45	1670.42	1	1670.42	189.86	< 0.0001
NaCl Conc., B	−12.65	1280.18	1	1280.18	145.50	< 0.0001
Incubation period, C	22	3872	1	3872	440.09	< 0.0001
Inoculum size, D	11.425	1044.24	1	1044.24	118.68	< 0.0001
Incubation temp., E	−4.45	158.42	1	158.42	18.00	0.0022
AB	1.38625	30.74	1	30.74	3.49	0.0944
AE	−1.48625	35.34	1	35.34	4.01	0.0760
BD	−2.71125	117.61	1	117.61	13.36	0.0053
BE	17.65125	4985.06	1	4985.06	566.60	< 0.0001
A^2^	−11.9997	3532.64	1	3532.64	401.52	< 0.0001
B^2^	−3.59973	317.90	1	317.90	36.13	0.0002
C^2^	−8.27473	1679.82	1	1679.82	190.92	< 0.0001
D^2^	−9.68723	2302.26	1	2302.26	261.67	< 0.0001
E^2^	−17.5497	7556.09	1	7556.09	858.82	< 0.0001
A^2^B	−7.42375	293.93	1	293.93	33.40	0.0003
A^2^C	−21.6013	2488.60	1	2488.60	282.85	< 0.0001
A^2^D	−8.23875	362.01	1	362.01	41.14	0.0001
A^2^E	−18.7013	1865.26	1	1865.26	212.00	< 0.0001
AB^2^	−13.8863	1028.41	1	1028.41	116.89	< 0.0001
Residual		79.18	9	8.79		
Lack of Fit		79.13	7	11.30	484.50	0.0021
Pure Error		0.04	2	0.02		
Total		38,449.44	*28*	*R*^2^ = 0.9979	Adj-*R*^*2*^ = 0.9935	

**Fig. 2:**
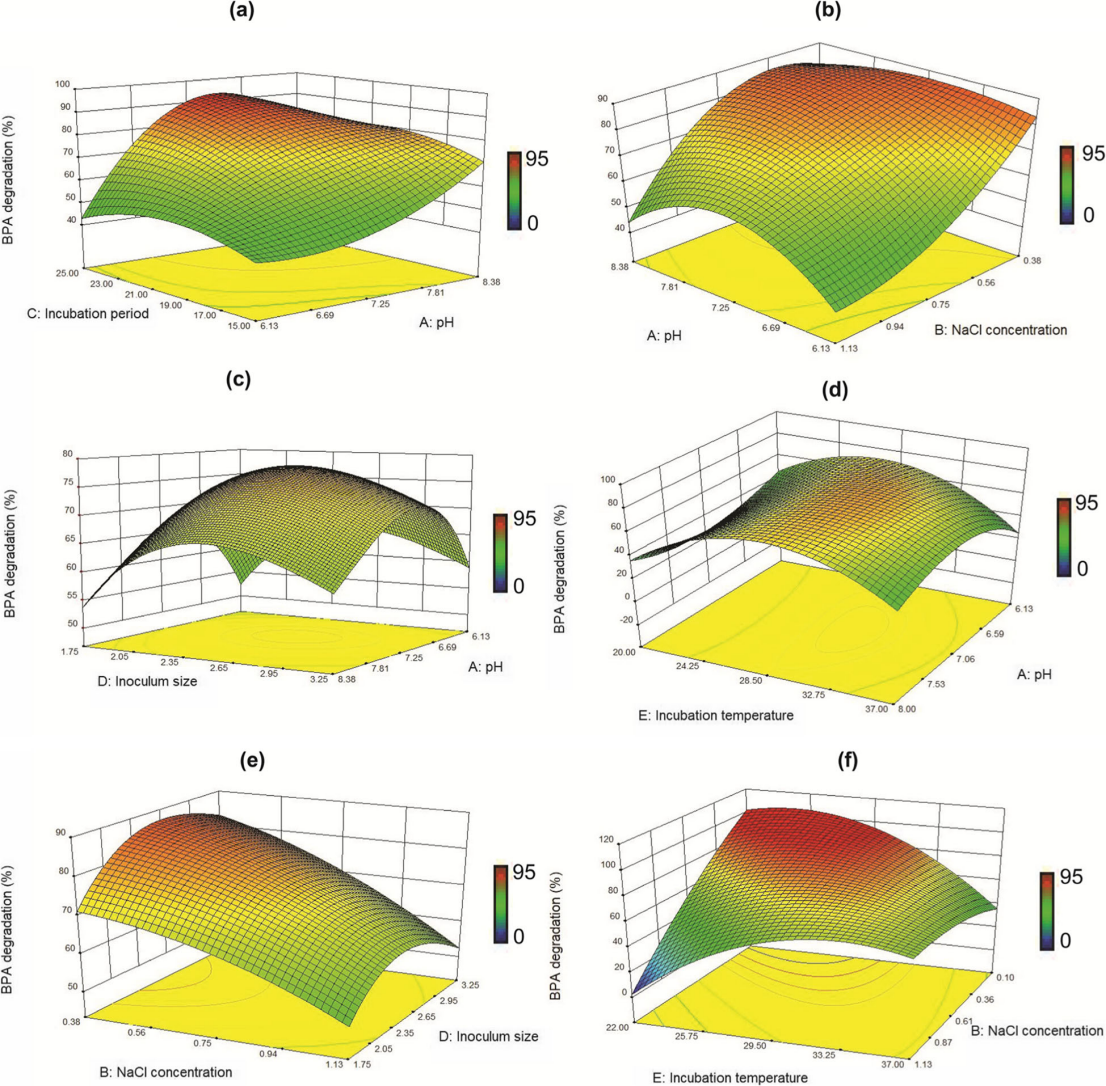
Response surface plots showing the effect of incubation period and pH (**a**); pH and NaCl concentration (**b**); inoculum size and pH (**c**); incubation temperature and pH (**d**); NaCl concentration and inoculum size (**e**); incubation temperature and NaCl (**f**)

To determine the optimum values of BPA degradation conditions for obtaining the highest and fastest degradation rate, three-dimensional (3-D) response surface curves were plotted as shown in Fig. 2. These results showed that an optimum was observed near the central value of pH, temperature and inoculum size. *Pseudomonas putida* YC-AE1 could grow and degrade BPA over a wide range of pH values with optimum value 7.2, Fig. 2a-d. The biodegradation of BPA was highly affected by the incubation period, it can be easily observed this effect from the high positive coefficient value (+ 22) of the variable as shown in Table 2. From Fig. 2a, prolonged incubation period was shown to increase the degradation of BPA by our strain YC-AE1.

The effect of temperature on BPA degradation by YC-AE1 showed in Fig. 2d. There was a gradual increase in BPA degradation by YC-AE1 by increasing the temperature from 15 to 25 °C and the maximum degradation was observed between 25 and 30 °C. The degradation then decreased beyond increasing temperature degrees. As shown in Table 2 the addition of NaCl to the degradation medium (TEM) has a reverse effect on BPA degradation, as described by a negative coefficient value (− 12). Data illustrated in Fig. 2b, e, f showed a sever decrease in BPA degradation by YC-AE1 with increasing NaCl concentration. The optimum inoculum size for BPA degradation was 2.5% and this can be clearly observed in the data plotted in Fig. 2c, e.

### Substrate utilization test

**Fig. 3:**
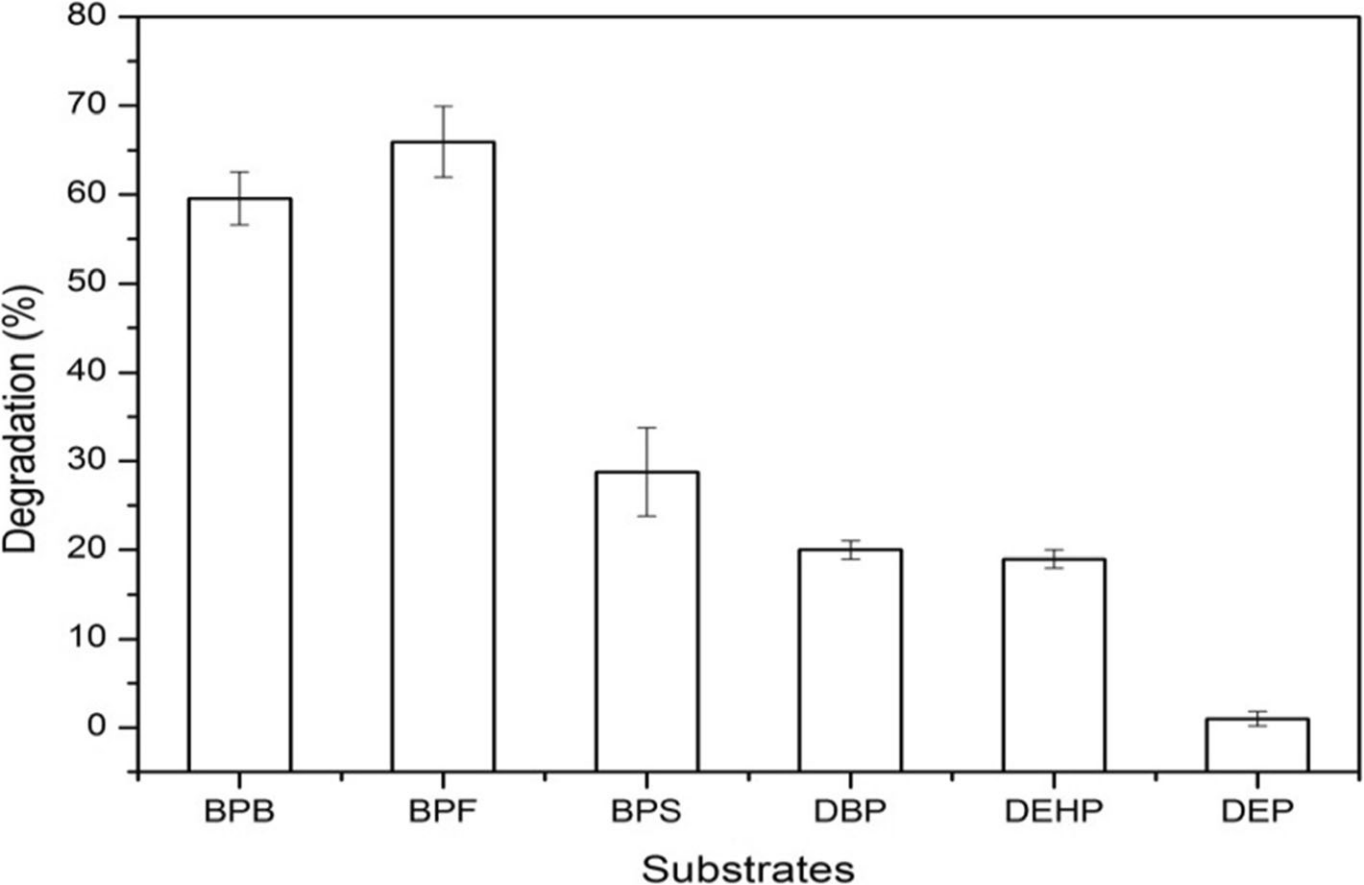
Degradation efficiency for different pollutants (100 mg l^−1^) by *Pseudomonas putida* YC-AE1. Error bars indicate the standard deviation obtained in three independent experiments

The capability of *Pseudomonas putida* strain YC-AE1 to degrade six different organic pollutants (BPB, BPF, BPS, DBP, DEP and DEHP) was examined. Data presented in Fig. 3 showed that, the YC-AE1 has an ability to degrade all examined pollutants in varying proportions. *Pseudomonas putida* YC-AE1 showed high ability to degrade about 60 and 67% of bisphenol B and F (100 mg l^− 1^), respectively, while lower degradation was observed in both bisphenol S, DBP and DEHP with about 30, 20 and 18% respectively.

### High and low BPA concentration for efficient biodegradation

**Fig. 4:**
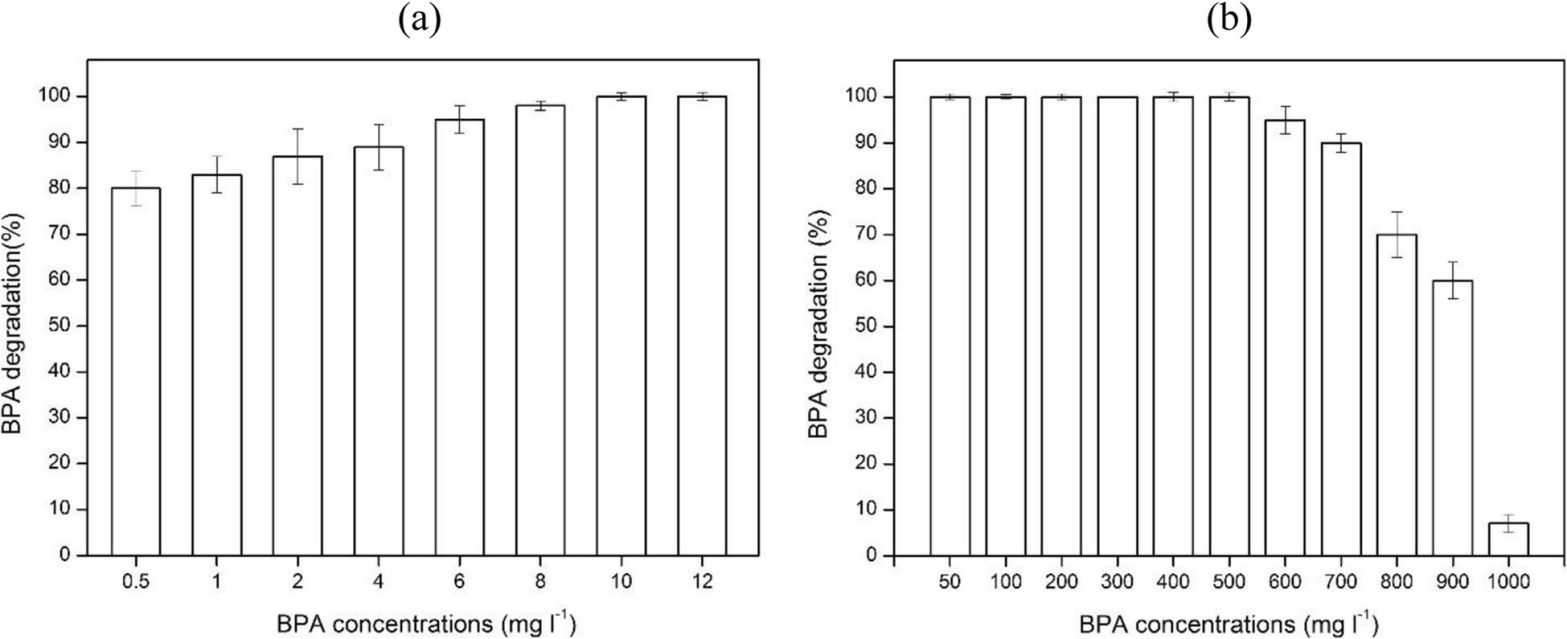
Ability of *Pseudoonas putida* YC-AE1 to degrade Low (**a**) and High (**b**) BPA concentrations. Error bars indicate the standard deviation obtained in three independent experiments

**Fig. 5:**
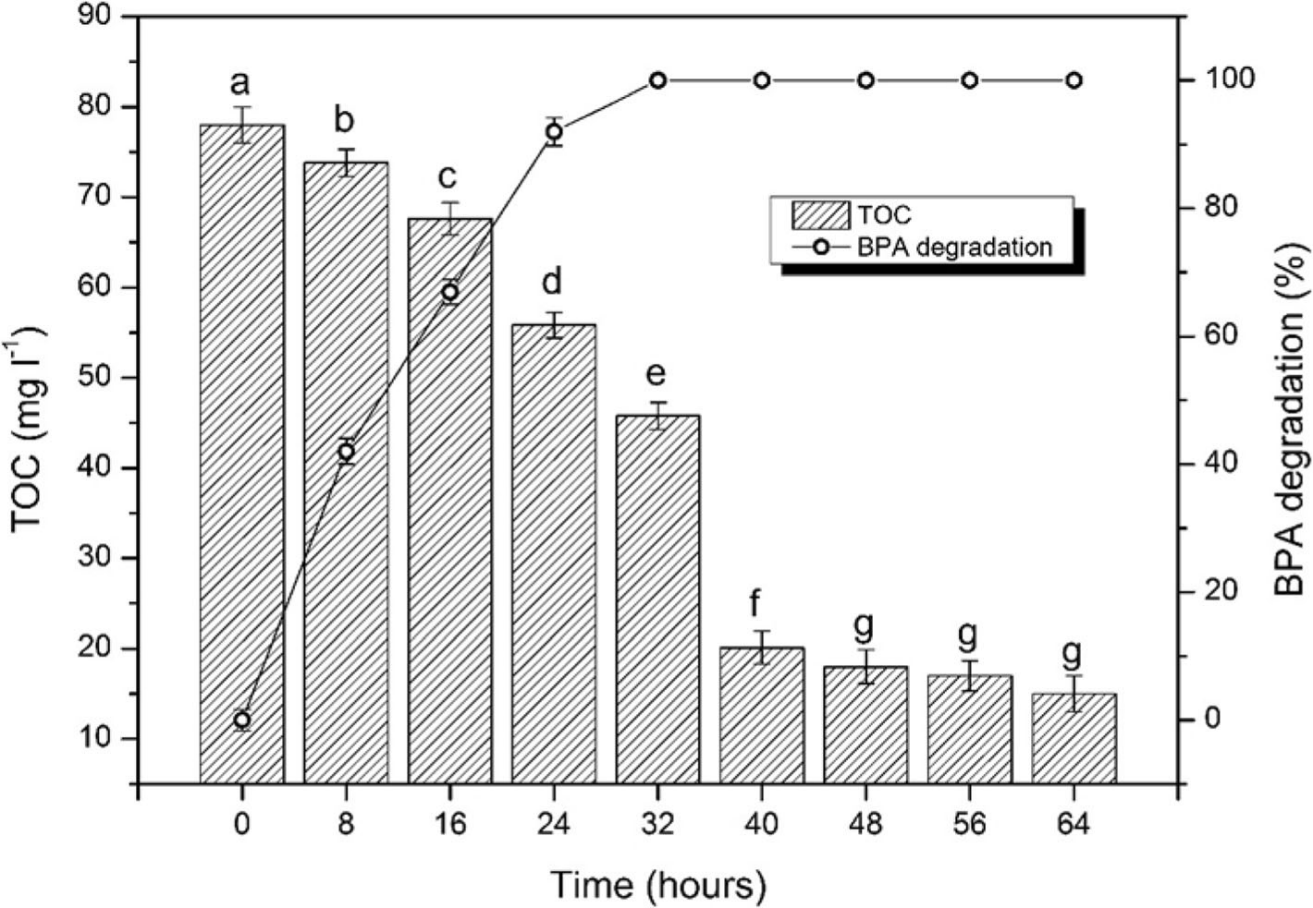
Total organic carbon depletion and BPA degradation by *Pseudomonas putida* YC-AE1. The different letters represent significant differences at *P* ≤ 0.05

The degradation rates in low and high BPA concentration are demonstrated in Fig. 4a, b after 15 and 72 h cultivation, respectively. As shown in Fig. 4a, the strain was able to degrade more than 80% of 0.5 mg l^− 1^ BPA then, the degradation gradually increased with increasing the concentration to reach 100% with 10 and 12 mg l^−^ in that short incubation time (15 h). On the other hand of high concentrations (Fig. 4b, the strain YC-AE1 was able to degrade 100% of BPA (50–500 mg l^− 1^). However, when the BPA concentration increased (600, 700, 800, 900 and 1000 mg l^− 1^) the degradation rate decreased to (95, 90, 70, 60 and 7%), respectively. Further incubation of strain YC-AE1 for another 2 days resulted in complete degradation of BPA (600–1000 mg l^− 1^). For detection of BPA mineralization, TOC experiment was performed, and the data obtained illustrated in Fig. 5. There was a dramatical significant decreasing in TOC starting from 78 to 20 mg l^− 1^ after 0 and 32 h incubation, respectively, (i.e., 75% depletion in TOC). With the degradation time further going on, there was non-significant decreasing in TOC to reach finally 82% at 64 h incubation.

### Metabolic intermediates and metabolic pathway

**Fig. 6:**
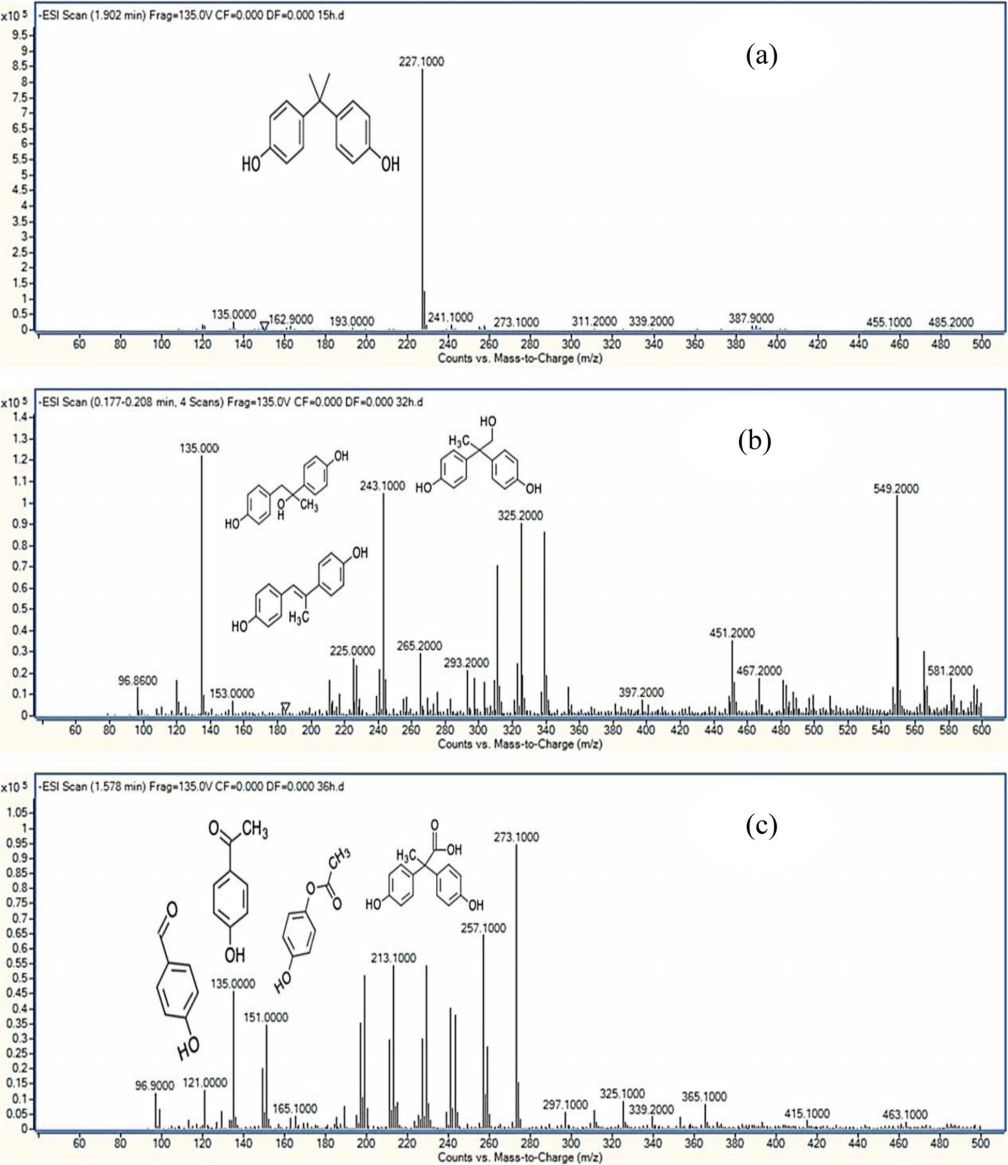
Mass spectra from HPLC-MS during BPA degradation by *Pseudomonas putida* YC-AE1 and detected intermediate compounds, BPA (**a**), 4,4-Dihydroxy-alpha-methylstilbene, 1,2-bis (4-hydroxyphenyl)-2-propanol and 2,2-bis(4-hydroxyphenyl)-1-propanol (**b**), 2,2-bis (4-hydroxyphenyl) propanoate, *p*-HBAL, *p*-HAP and HPA (**c**)

**Fig. 7:**
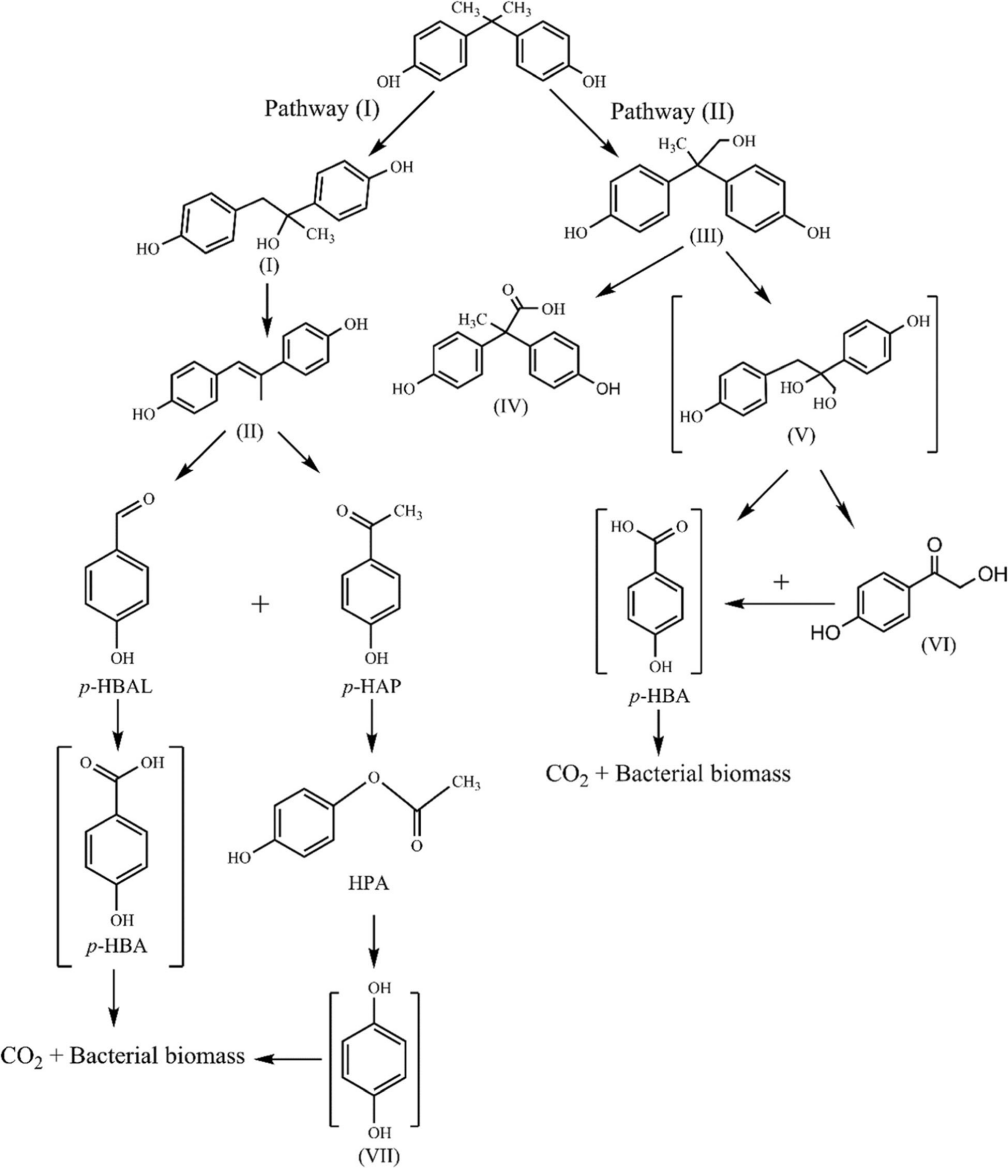
Proposed pathway for BPA degradation by *Pseudomonas putida* YC-AE1, 1,2-bis(4-hydroxyphenyl)-2-propanol (I), 4,4-dihydroxy-alpha-methylstilbene (II), 2,2-bis(4-hydroxyphenyl)-1-propanol (III), 2,2-bis (4-hydroxyphenyl) propanoate (IV), 2,3-bis(4-hydroxyphenyl)-1,2-propanediol (V), 4-hydroxyphenacyl alcohol (VI), hydroquinone (HQ) (VII). The compounds in parentheses were not detected in our study but assumed to be produced

Detection of metabolic intermediates during BPA degradation with optimum conditions (200 mg l^− 1^ BPA, pH 7.2, 30 °C and 2.5% inoculum size) was performed. Based on the mass spectra (LC-MS) as shown in Fig. 6, eight compounds relating to BPA degradation were identified as following, BPA (m/z 227.1), 4,4-Dihydroxy-alpha-methylstilbene (m/z 225), *p*-hydroxybenzaldeyde (*p*-HBAL) (m/z 122), *p*-hydroxyacetophenone (*p*-HAP) (m/z 136), 4-hydroxyphenylacetate (HPA) and 4-Hydroxyphenacylalcohol (same m/z, 152), 2,2-bis(4-hydroxyphenyl)-1-propanol and 1,2-bis (4-hydroxyphenyl)-2-propanol (same m/z, 244) and 2,2-bis (4-hydroxyphenyl) propanoate (m/z 258). Based on the intermediates obtained during the biodegradation of BPA, the proposed pathway for BPA biodegradation by strain YC-AE1 is represented in Fig. 7. There were two different proposed pathways for BPA degradation by YC-AE1, both of them start by hydroxylation for BPA to form 1,2-Bis(4-hydroxyphenyl)-2-propanol and 2,2-Bis(4-hydroxyphenyl)-1-propanol in pathway (I) and (II), respectively. In pathway (I), 1,2-Bis(4-hydroxyphenyl)-2-propanol dehydrated to 4,4-Dihydroxy-alpha-methylstilbene [32]. The previously mentioned compound was further oxidized to form *p*- HBAL and *p*- HAP. *p*- HAP was metabolized to HPA and then to HQ. Both of hydroxybenzoic acid (HBA) and HQ were assumed mineralized to carbon dioxide (CO_2_) and bacterial biomass through benzoate degradation pathway. HBA and HQ were not detected and presumed in the pathway. In pathway (II), 2,2-Bis(4-hydroxyphenyl)-1-propanol is metabolized to form 2,2-bis (4-hydroxyphenyl) propanoate and 2,3-Bis(4-hydroxyphenyl)-1,2-propanediol. The intermediate (2, 3-Bis (4-hydroxyphenyl)-1,2-propanediol) was further metabolized through many steps (as shown in Fig. 7) to form finally HBA which mineralized to CO_2_ and bacterial biomass through benzoate degradation pathway.

## Discussion

There currently is increasing interest in endocrine disruptors, and their biodegradation by bacteria has been extensively studied. BPA is a known endocrine disruptor that displays estrogenic activity and acute toxicity [3]. Finding new bacterial strains with strong degradation capacity and prominent adaptability towards a wide range of environmental conditions is of great importance. In our study, one bacterial strain showed excellent biodegradation performance was isolated and identified as *Pseudomonas putida* strain YC-AE1 according to identification methods. YC-AE1 could degrade 200 mgl^− 1^ within 20 h under optimum environmental factors. For optimizing the factors affecting the biodegradation process, RSM was performed using CCD. RSM is used to determine the combined effect of several variables and their interactions. A statistical optimization approach using CCD was used to study the linear, quadratic and interactive effects of various parameters on BPA degradation by *Pseudomonas putida* strain YC-AE1. The F-value and probability value (*p*-value) are tools for evaluating the significance of each of the parameters in the model equation. The pattern of interactions between the variables is indicated by these coefficients. The larger F- value and the smaller *P*-value are an indication of the high significance of the corresponding coefficient [30]. The high value of R^2^ (so close to 1) supports the accuracy of the model and demonstrates a good correlation between actual and predicted values of BPA degradation. The significance of the model and high R^2^, support the model to predict responses. For microbial effective process, it is necessary to monitor and control parameters that affect the process like, inoculum volume, salt concentration, pH, temperature, incubation period, etc. Maximum BPA degradation was observed at pH of 7.2 and our results was different from that obtained by [25, 33] which reported that maximum BPA degradation was shown at pH 7 by *Achromobacter xylosoxidans* strain B-16 and *Arthrobacter* sp. YC-RL1.

The inoculums to be used in degradation medium must be in a healthy and active state moreover being of optimum size. Such conditions possibly minimize the length of the log phase. Lower inoculum volumes decrease the degradation efficiency of YC-AE1 and, this is may be due to low number of bacterial cells that decrease the amount of enzymes for biodegradation. Also, inoculum size higher than the optimum value (2.5%) had a little increasing effect on BPA degradation by YC-AE1, and this may be because the reduction of dissolved oxygen and increased competition towards nutrients [34]. Zhang et al. [25] reported that, increasing the size of inoculum leads to increase the degradation of BPA by *Achromobacter xylosoxidans* strain B-16 isolated from compost leachate of municipal solid waste.

Temperature is one of the most important parameters that affect any microbial process. The growth rate of microorganisms becomes slow below or above the optimum growth temperature because of a reduced rate of cellular production [35]. Enzyme thermal stability and activity is correlated to an organism’s growth temperature and also, degradation is an enzyme-controlled activity hence as the temperature increases, the cellular growth and physiological functions increase to an optimum value [36]. Fouda [37] reported a relatively high optimum temperature (35–40 °C) for BPA biodegradation by *Klebsiella pneumoniae* J2 and *Enterobacter asburiae* L4. The biodegradation of BPA was greatly reduced by addition of NaCl to the medium component and this result may be due to the effect of NaCl on the growth curve of our strain by elongating the lag phase and delaying the degradation process [38, 39] and this explains why the strain regains its ability to degrade BPA in the presence of salt by further incubation for another 2 days resulted in degradation of 70% of BPA (Data not shown).

It is very important for bacterial strain intended to use in natural remediation to has ability to degrade many pollutants or at least many derivatives of one pollutant. For that purpose, our strain *Pseudomonas putida* YC-AE1 was examined to degrade and tolerate 6 different BPA-related organic pollutants. BPS was the hardest one in the examined bisphenols to degrade because it contains S=O double chemical bond, which gives the structure chemical durability. The steric effects between the substrates and the responsible enzymes declines from BPB to BPF, which may be the reason why BPF is easier to degrade. The lowest degradation ability was shown with DEP with degradation rate not more than 3%. Many Pollutants were reported to be degraded by *Pseudomonas sp* by researchers. For example, phenols [40], phenolics like pentachlorophenol [41] and Catechol [42]. Under normal environmental conditions, the concentration of environmental pollutants is always very low compared with that examined in vitro [43, 44] and these pose many problems for bioremediation process because, the concentration of pollutants is too low to sustain microbial growth and ensure the accessibility to microbe and even more than that to induce metabolic genes [33]. Therefore, determining the survival ability of *Pseudomonas putida* YC-AE1 against both high and low BPA concentrations is important. Strain YC-AE1 could survive in both conditions and remain active in all BPA concentrations. This performance and ability of strain YC-AE1 to degrade and tolerate these extremely low and high concentrations are important for application in BPA bioremediation especially with fast degradation rate (200 mg l^− 1^ in 20 h). This quality makes strain YC-AE1 a promising bacterium compared with other reported strains. Suyamud et al. [27] reported *Bacillus megaterium* strain ISO-2, which can degrade 5 mg l^− 1^ of BPA within 72 h on mineral salt medium supplemented with yeast extract as co-substrate. *Sphingomonas bisphenolicum* strain AO1 was reported to degrade 100 mg l^− 1^ BPA to undetectable level within 48 h in minimum medium with 1% glucose [28].

Biodegradation of BPA and its metabolic intermediates could not fully support the mineralization of BPA, TOC experiment could show the mineralization rate of BPA. Although, faster BPA mineralization rate has generally been reported for microbial communities than the culture of BPA-degrading lonely strains [45], interestingly, our strain *Pseudomonas putida* YC-AE1 lonely demonstrated high BPA mineralization compared with microbial communities. Although, the TOC depletion percentage in our experiment (82% after 64 h) by *Pseudomonas putida* YC-AE1 was closely similar to that reported by Yu et al. [45] that used co-culture of *sphingomonas* sp. (Sph-2) and *Pseudomonas* sp. (84% after 72 h), Surprisingly, our experiment was conducted with 100 mg l^− 1^ compared with 50 mg l^− 1^ BPA by Yu et al. [45], and these results reflect the high degradation and mineralization rate of our strain YC-AE1.

The intermediates detected in our study have a good consistency with those reported by Eio et al. [32]. Das et al. [46] reported many different intermediate compounds detected during BPA degradation by recombinant laccase from *Bacillus* sp. GZB. Three different pathways were proposed based on three intermediates, Hydroxybenzaldeyde, *p*-hydroxybenzoic acid and *p*-hydroquinone (HQ) detected during biodegradation of BPA by *Achromobacter xylosoxidans* strain B-16 isolated from compost leachate [25]. Based on the intermediates obtained in our study using LC-MS and in light of literature [25, 27, 32, 46, 47], two degradation pathway by *Pseudomonas putida* YC-AE1 was proposed. The intermediates were detected from both two degradation pathways at same experiment and this is an evidence that our strain use both of pathways in the degradation and this may interpret the high degradation rate of YC-AE1.

## Conclusion

This study has reported the ability of promising *pseudomonas putida* strain YC-AE1 isolated from Guangdong province (China) to degrade BPA as the sole source for carbon with excellent adaptation to a wide range of environmental conditions. This strain can degrade both of extremely high and low concentrations of BPA and mineralize it in a short time compared with reported strains. Furthermore, it can degrade many BPA-related pollutants. Analysis of intermediates showed two degradation pathways for BPA which may interpret the fast degradation rate of our strain. These criteria recommend *pseudomonas putida* YC-AE1 as a promising strain for natural remediation of environmental pollutant BPA. Also, our study provides a good microbial source for further studies on bacterial BPA degradation metabolism and molecular mechanism responsible for that degradation.

## Methods

### Chemicals and medium

The Bisphenols (BPA, BPB, BPF and BPS), DBP, DEHP and DEP used in this study were purchased from Shanghai Macklin Biochemical Co., Ltd. China, (all purities above 99%). Stock solutions (20,000 mg l^− 1^) of all these substrates were prepared by dissolving each individual in methanol (99%). Other chemicals used were of analytical reagent grade, all solvents used were high performance liquid chromatography (HPLC) grade. Two types of media were used in this study: (1) Luria-Bertani (LB) medium for growth and enrichment. It consisted of 10.0 g l^− 1^ peptone, 5.0 g l^− 1^ yeast extract, and 10.0 g l^− 1^ NaCl. (2) Trace Elements Medium (TEM) as described by [48] were used for isolation of BPA biodegradable strains and statistical optimization experiments.

### Isolation, screening and culture conditions for high tolerant BPA degrading strain

Soil and water samples were collected from five different provinces in China and these provinces were Heilongjiang, Hebei, Shandong, Guangdong and Anhui. Five grams or 5 ml of soil or water samples respectively, were separately added to 100 ml of TEM in 250 ml Erlenmeyer flask and supplied with 100 mg l^− 1^ of BPA. The flasks were incubated in a rotary shaker at 30 °C with 180 rpm. After 7 days, 5 ml of the culture (each flask) was transferred to fresh TEM with 200 mg l^− 1^ and incubated at the same condition mentioned above for 7 days. This process was repeated three more times with increasing BPA concentrations in each run to reach 500 mg l^− 1^ [49]. The final cultures were spread on solid TEM containing 100 mg l^− 1^ BPA as a sole source of carbon and incubated for 5 days at 30 °C, single colonies were picked and inoculated into TEM supplemented with 100 mg l^− 1^ BPA and incubated (30 °C and 180 rpm) to test the biodegradation. Uninoculated fresh culture of TEM with 100 mg l^− 1^ BPA was set as abiotic control. The residual concentrations of BPA were determined by using HPLC.

### Identification of BPA degrading strain

The total genomic DNA of bacterial isolate was extracted by using Bacterial Genomic Extraction Kit (Takara, Japan). The 16S rRNA gene was amplified from genomic DNA by using two universal primers (**27F**: AGAGTTTGATCCTGGCTCAG, **1492R**: AAGGAGGTGATCCAGCC). The PCR product was sequenced (Sangon Biotech Co., Ltd., Beijing, China) and the obtained sequence was deposited to GenBank, and aligned to get the similar sequences by BLAST (https://blast.ncbi.nlm.nih.gov/Blast.cgi). The phylogenetic tree was constructed using MEGA 5 software [50] with a neighbor-joining algorithm [51]. The tree was drawn to scale, with branch lengths in the same units as those of the evolutionary distances used to infer the phylogenetic tree. The evolutionary distances were computed using the maximum composite likelihood method [52]. To confirm 16S rRNA gene identification and determining the biochemical tests of the bacterial isolate, Biolog tests (BIOLOG inc., USA), was accomplished and the results were analyzed by Microlog 3 (version 5.2) software.

### Statistical optimization of BPA degradation by response surface methodology (RSM)

RSM was conducted using a factorial central composite design (CCD). CCD is one of the most common design used in RSM which has the same predictability in all directions from the center [53]. Five experimental factors selected to design the experiment included pH (A), NaCl concentration (B), incubation period (C), inoculum size (D) and incubation temperature (E) with 5 levels for each factor, generating 29 trials using Design-Expert software (version 8). The Reduced Cubic Model was fitted for biodegradation response analysis. The value of the dependent response was the mean of three replications. The detailed experimental designs of five parameters are shown in Table 1. In this experiment, TEM with BPA (200 mg l^− 1^) as sole source of carbon was used and inoculum was prepared by growing the bacterial strain on L. B medium to reach (O.D_(600)_ = 0.8), the bacterial cells were collected by centrifugation at 5000 rpm for 5 min and washed twice by fresh TEM and resuspended using the same original volume with TEM.

### Substrate utilization test

To determine the ability of selected strain to degrade different organic pollutants other than BPA, the bacterial strain was inoculated (1% inoculum size) into TEM (15 ml) supplemented with 100 mg l^− 1^ of each of the following substrates: bisphenol B (BPB), bisphenol F (BPF), bisphenol S (BPS), Dibutyl phthalate (DBP), Diethylhexyl phthalate (DEHP) and Diethyl phthalate (DEP). The cultures were incubated in a rotary shaker (30 °C and 180 rpm) for 72 h. Cultures without inoculation were set as abiotic control. The residual concentrations of bisphenols (B, F, and S) and (DBP, DEHP) were measured by HPLC and gas chromatography (GC) respectively.

### Detection of intermediates

To determine the biodegradation intermediates, the selected bacterial strain was grown in TEM supplemented with BPA (200 mg l^− 1^) under optimal culture conditions. 100 ml of sample was taken from the culture at specific intervals as following (15, 20, 24, 28, 32, 36 h) and centrifuged at 10000 rpm for 10 min, the cell-free supernatant was acidified (pH: 2–3) using HCl. The samples were extracted twice with the same volume of ethyl acetate, organic layer separated and dried by Rotary evaporator (BUCHI, Switzerland) and re-suspended in 1 ml methanol (99%). The samples were subjected to high performance liquid chromatography-mass spectroscopy (HPLC-MS) to detect the intermediates.

### BPA degradation at low and high concentrations and total organic carbon (TOC) determination

The concentration of BPA in natural polluted environments is very low compared with that always examined in vitro. Therefore, the ability of bacterial strain to degrade and utilize BPA at low and high concentrations is a very important character especially in strains intended to use in environmental bioremediation. The high and low concentrations used were as follows (50, 100, 200, 300, 400, 500, 600, 700, 800, 900, and 1000 mg l^− 1^) and (0.5, 1, 2, 4, 6, 8, 10 and 12 mg l^− 1^) respectively. The residual BPA concentration and degradation rate were determined after 15 and 72 h for low and high concentrations by HPLC, respectively. In low concentration experiment, the relatively short incubation time was to detect the differences in degradation rates compared with BPA concentrations. To detect the BPA mineralization rate, TOC depletion was determined during the degradation time. The selected bacterial strain was grown in TEM supplemented with 100 mg l^− 1^ of BPA at 30 °C. Samples (20 ml) were taken in triplicate each 8 h intervals as following (8, 16, 24, 32, 40, 48, 56 and 64 h) and centrifugated at 15000 rpm for 30 min to eliminate the bacterial cells. The cell-free supernatant was kept at − 20 °C. TOC was determined for all preserved samples by Walkley-Black method [54].

### Analysis methods

Cell growth was measured as optical density (OD) at 600 nm using a UV-Visible spectrophotometer (Thermo-Scientific, USA). For detection of bisphenols, an Agilent 1200 system HPLC (Agilent, USA) with visible ultraviolet detector was used equipped with Eclipse XDB C18 column (4.6 × 150 mm × 5 μm). The mobile phase was acetonitrile 85% and (water, 0.1% acetic acid) 15%, with a flow rate 1 ml/minute and detecting wavelength 220 nm. The retention time was 1.55 min and 2 μl for sample injection, the detection limits were 0.3, 0.4, .4, 0.5 μgml^− 1^ for BPA, BPB, BPF and BPS, respectively. For detection of DBP, DEHP and DEP, a GC system (GC-2010 SHIMADZU, Kyoto, Japan) with a flame ionizing detector (FID) was used. Samples were extracted twice by an equal volume of n-hexane and the hexan phase was separated and analyzed by GC [55], the detection limits were 0.1 μgml^− 1^. The intermediate metabolites of BPA degradation were analyzed by (HPLC–MS, 1290, USA). The mobile phase was a mixture of (50:50%) of water and acetonitrile (with 0.1% formic acid for each one), running at a flow rate of 1 mL/min. The sample was injected (2 μL) as methanolic extract and scan mode with 3 kV capillary voltage was used to analyze negative ionization. The dry gas flow was 5 L/min and the dry nitrogen was heated to 325 °C. Mass hunter (version A.02.00, Agilent, USA) was used to analyze and collect the data.

## Data Availability

All data generated or analyzed during this study are included in this published article except the sequence of 16S rRNA gene was deposited to Gen Bank (NCBI) with accession number, MK318658.

## Abbreviations

BPA: Bisphenol A
BPB: Bisphenol B
BPF: Bisphenol F
BPS: Bisphenol S
CCD: Central composite design
DBP: Dibutyl phthalate
DEHP: Diethylhexyl phthalate
DEP: Diethyl phthalate
GC: Gas chromatography
HPA: Hydroxyphenylacetate
HPLC: High performance liquid chromatography
HQ: Hydroquinone
*p*-HAP: *p*-hydroxyacetophenone
*p*-HBAL: *p*-hydroxybenzaldeyde
RSM: Response Surface Methodology
TEM: Trace elements medium
TOC: Total organic acrbon
